# Cord Blood Mitochondrial DNA Copy Number and Physical Growth in Infancy and Toddlerhood: A Birth Cohort Analysis

**DOI:** 10.3390/children12101369

**Published:** 2025-10-10

**Authors:** Hisanori Fukunaga, Takeshi Yamaguchi, Hiroyoshi Iwata, Atsuko Ikeda

**Affiliations:** 1 Faculty of Health Sciences, Hokkaido University, N12 W5 Kita-ku, Sapporo 060-0812, Japan; atsuko_ikeda@hs.hokudai.ac.jp; 2 Center for Environmental and Health Sciences, Hokkaido University, N12 W7 Kita-ku, Sapporo 060-0812, Japan; takeshi7698@med.hokudai.ac.jp (T.Y.); hiroyoshi-iwata@cehs.hokudai.ac.jp (H.I.)

**Keywords:** cord blood, DNA copy number variation, DOHaD, physical growth, mitochondrial DNA

## Abstract

**Background/Objectives:** Cord blood mitochondrial DNA copy number (mtDNAcn) has been proposed as a biomarker reflecting environmental influences during fetal life, with reported associations with perinatal outcomes such as birth weight and length. Within the framework of the Developmental Origins of Health and Disease (DOHaD) theory, this study aimed to investigate whether cord blood mtDNAcn is related to postnatal physical growth in early childhood. **Methods:** We analyzed data from 150 newborns (68 females and 82 males) enrolled in the Tohoku Medical Megabank Birth and Three-Generation Cohort Study in Japan. Cord blood mtDNAcn was quantified using real-time PCR, and standard deviation scores for weight and height were assessed at 1, 2–3, 4–6, 18–24, and 36–48 months of age. Correlation analyses were conducted separately by sex. **Results:** Cord blood mtDNAcn showed no significant associations with body weight or height at any of the postnatal time points up to 48 months of age. Growth trajectories of infants with higher or lower mtDNAcn values at birth tended to converge toward the population mean during infancy and toddlerhood. **Conclusions:** Although no significant relationships were observed, this exploratory, hypothesis-generating study provides a foundation for future investigations. Larger cohorts with extended follow-up are needed to clarify the potential significance of cord blood mtDNAcn in early-life research on child growth and health.

## 1. Introduction

Mitochondrial DNA (mtDNA) and nuclear DNA (nDNA) cooperate closely to orchestrate the molecular processes underlying cellular respiration in eukaryotic cells [1]. Human mtDNA is a 16,569 base-pair cyclic multi-copy genome [2], typically with tens to thousands of copies per cell. Although mtDNA is considerably smaller than nDNA in terms of genomic size, it plays a disproportionately large role in cellular transcriptional output; in the heart, for example, transcripts derived from mtDNA account for approximately 30% of total mRNA, while in other organs, the proportion ranges from 5% to 25% [3]. Alterations in mtDNA, including point mutations, deletions, and reductions in copy number, have been associated with mitochondrial dysfunction. Thus, the proper control of mtDNA copy numbers (mtDNAcn) is essential not only at the cellular level but also at the individual level [4].

According to prior research analyzing the relationship between blood-derived mtDNAcn and gene expression across 47 tissues in 419 individuals, mtDNAcn in peripheral blood serves as a biomarker reflecting systemic metabolic health [5]. Furthermore, previous studies have demonstrated that mtDNAcn in peripheral blood decrease with age [6,7] and are negatively associated with all-cause mortality [8,9]. Blood-derived DNAcn has also been reported to be associated with neurodegenerative diseases [10], diabetes [11], and cancer [12]. Thus, mtDNAcn in peripheral blood possibly serves as a biomarker of health and disease.

The Developmental Origins of Health and Disease (DOHaD) theory proposes that environmental exposures during critical periods of early development may influence health outcomes and disease susceptibility across the life course [13,14,15]. Accumulating evidence from both epidemiological investigations and animal studies indicates that environmental exposures during the embryonic, fetal, neonatal, and early childhood periods are linked to health outcomes across the lifespan, including the development of chronic diseases in adulthood and later life. Epigenetic alterations have emerged as key mediators in the mechanisms underlying the effects proposed by the DOHaD hypothesis. From the perspective of DOHaD theory, cord blood mtDNAcn has been considered one of the indicators of environmental influences during fetal life, as well as a surrogate marker for predicting health risks in adulthood [16]. Supporting this view, a recent longitudinal study from the Columbia Children’s Center for Environmental Health (CCCEH) Mothers and Newborns Cohort, which recruited women between 1998 and 2006 from Northern Manhattan and the South Bronx, New York, USA, demonstrated that mtDNAcn dynamics from birth through adolescence were associated with several prenatal environmental factors, including prenatal environmental tobacco smoke exposure, maternal educational attainment (completion of high school), receipt of public assistance at birth, and maternal nativity outside the United States, further highlighting the sensitivity of mtDNAcn to intrauterine conditions [17]. This notion is further supported by a recent review that systematically evaluated studies linking a wide range of prenatal environmental exposures, including maternal smoking, psychosocial stress, dietary factors, and chemical pollutants, to variations in maternal and child mtDNAcn and mtDNA methylation. The review emphasized that such exposures may disrupt mitochondrial biogenesis and epigenetic programming during development, highlighting the susceptibility of mtDNAcn to intrauterine environmental influences [18].

Birth cohort studies are particularly well-suited for epidemiological research aimed at demonstrating the DOHaD theory. We previously reported mtDNAcn based on cord and peripheral blood from 149 three-generation families (*n* = 1041), consisting of newborns, mothers, fathers, maternal grandmothers, maternal grandfathers, paternal grandmothers, and paternal grandfathers and analyzed maternal factors during pregnancy and neonatal outcomes [19]. All participants were enrolled in the Tohoku Medical Megabank Project Birth and Three-Generation Cohort Study (TMM BirThree Cohort Study) in Japan [20], which has been conducted by the Tohoku University Tohoku Medical Megabank Organization (ToMMo) and Iwate Medical University Iwate Tohoku Medical Megabank Organization (IMM). We found that cord blood mtDNAcn was negatively correlated with perinatal outcomes, such as gestational age, birth weight, and umbilical cord length. However, because the analysis was cross-sectional, it remained unclear whether cord blood mtDNAcn was associated with long-term physical growth in early life.

The aim of the present study was to assess whether cord blood mtDNAcn at birth is associated with physical growth in infancy and toddlerhood. We examined 150 newborns (68 females and 82 males, including twins) enrolled in the TMM BirThree Cohort Study. By linking molecular markers such as mtDNAcn with longitudinal growth data, the study seeks to clarify the biological and environmental factors that shape child health and development.

## 2. Materials and Methods

### 2.1. Information and Biological Samples

We examined the relationship between cord blood mtDNAcn and physical growth during infancy and toddlerhood in a cohort of 150 newborns. All biospecimens and related data were provided by ToMMo and IMM. This study was approved by the Sample and Data Access Committee of the ToMMo and IMM (reference no. 2021-0021-1). The participants were primarily healthy at enrollment, with no known congenital disorders. Although some maternal conditions such as hypertension were present, the newborns themselves were considered generally healthy. Maternal and perinatal characteristics, including maternal age, body mass index, and smoking status, as well as the associations between maternal lifestyle factors—such as folic acid supplementation and exercise habits during pregnancy—and neonatal cord blood mtDNAcn, have been described previously for this cohort [19]. Information on infant feeding practices, including breastfeeding, was not available.

Trained genome medical research coordinators provided information on the TMM BirThree Cohort Study to potential participants and received a signed informed consent form from each participant. All the participants were recruited in the Tohoku region (in northeastern Japan) between 2013 and 2017. The participants in the TMM Project are still under follow-up and expected to provide further epidemiological data on growth; biological aging; and the incidence of various diseases, including lifestyle-related diseases [21]. Participation in the present study was based on an opt-out consent procedure, in accordance with institutional and ethical guidelines.

### 2.2. Determination of Mitochondrial DNA Copy Number

Cord blood DNA was extracted from buffy coat samples. As previously described [19], the mtDNAcn of the DNA samples was determined using the Applied Biosystems’ StepOne Real-Time PCR analysis (Thermo Fisher Scientific Inc., Waltham, MA, USA) and Human Mitochondrial DNA Monitoring Primer Set with TB Green^®^ Premix Ex Taq™ II (Tli RNase H Plus), ROX Plus (Takara BIO Inc., Shiga, Japan). The thermal profile was as follows: 2 min at 98 °C, 40 cycles at 98 °C for 10 s, 15 s at 60 °C, and 30 s at 68 °C.

To measure mtDNAcn relative to nDNA, the primer set contained two primer pairs each for detecting mtDNA and nDNA, for a total of four primers. These primers target two genes on mtDNA (*ND1*, *ND5*) and two genes on nDNA (*SLCO2B1*, *SERPINA*). Relative mtDNAcn was calculated using the ΔCt method, where the difference in threshold cycle (Ct) values between mtDNA and nDNA targets was used as an index of mtDNAcn.

As determined earlier [19], the mean ± standard deviation (SD) for cord blood mtDNAcn among the female and male newborns included in this study was 156.48 ± 64.9 and 180.5 ± 66.6, respectively, showing that cord blood mtDNAcn values were significantly lower among females than males (*p*  =  0.047, Mann–Whitney test).

### 2.3. Standard Deviation Calculation

We used data on standardized anthropometric values at birth by gestational age for Japanese infants (http://jspe.umin.jp/medical/keisan.html (accessed on 11 September 2025)) to calculate SD scores for weight and height in infancy and toddlerhood. Weight-for-age and height-for-age were measured at 1, 2–3, 4–6, 18–24, and 36–48 months.

As shown in Figures S1 and S2, we tracked changes over time in weight-for-age and height-for-age in the low (10th percentile or lower) and high (90th percentile or higher) mtDNAcn newborn groups. The former consisted of 8 females and 7 males, while the latter consisted of 8 females and 9 males. All children were in good health and had no notable medical history.

### 2.4. Statistical Analysis

**Figure 1:**
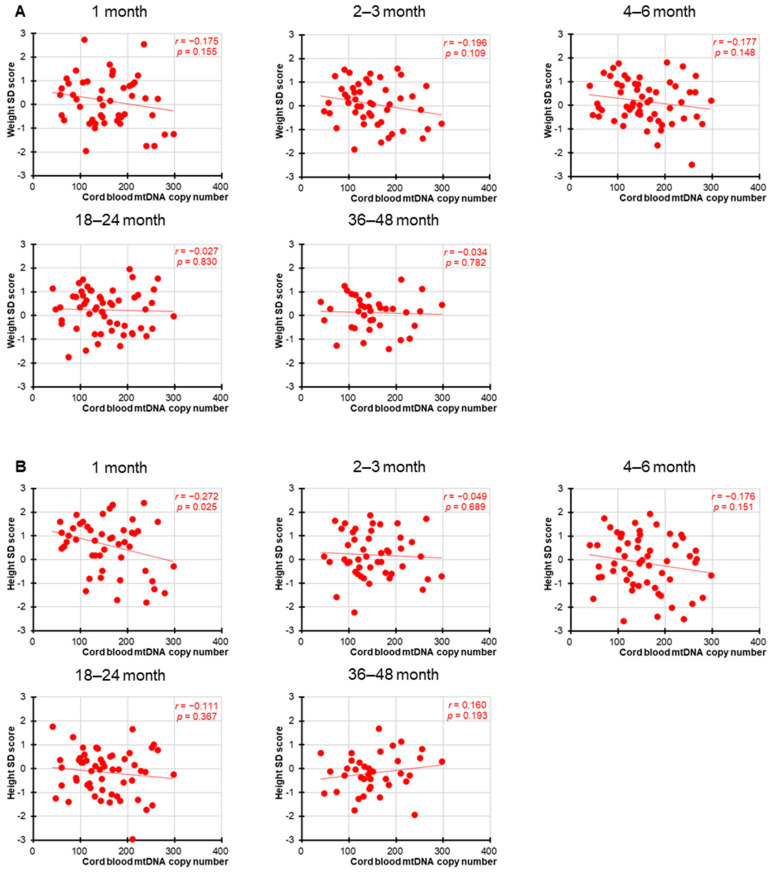
Correlation between female cord blood mtDNAcn and weight and height in infancy and toddlerhood. Correlation between female cord blood mtDNAcn and weight (**A**) and height (**B**) 1, 2–3, 4–6, 18–24, and 36–48 months after birth. The circles indicate individual sample values, and the straight line represents the correlation line. As 10 outcome measures were tested against one hypothesized predictor, to account for the increased possibility of type-I errors due to multiple testing, a Bonferroni-adjusted significance level of 0.005 (=0.05/10) was calculated.

**Figure 2:**
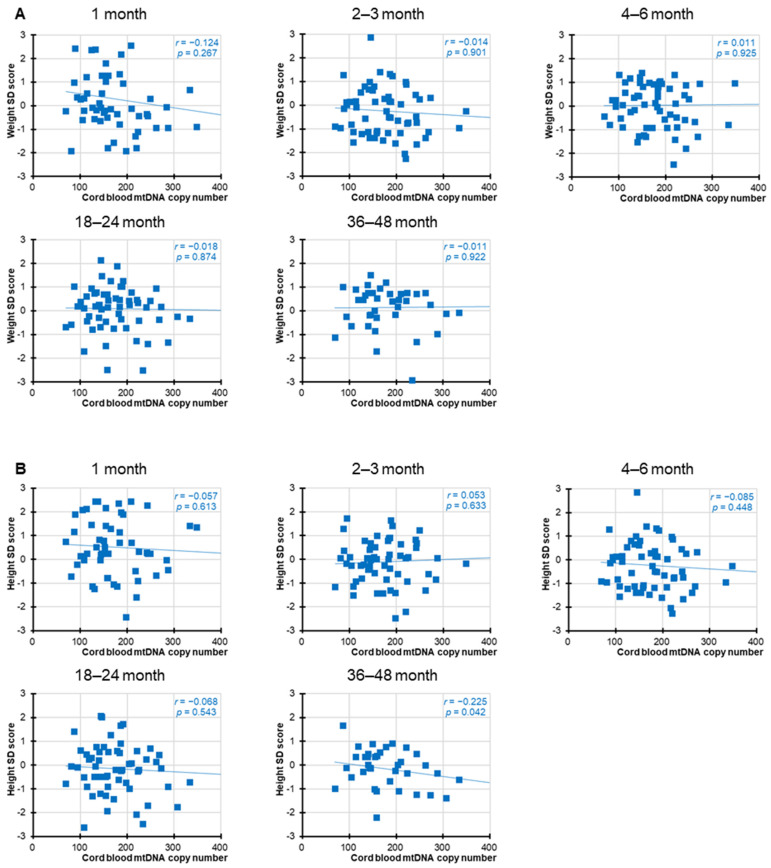
Correlation between male cord blood mtDNAcn and weight and height in infancy and toddlerhood. Correlation between male cord blood mtDNAcn and weight (**A**) and height (**B**) 1, 2–3, 4–6, 18–24, and 36–48 months after birth. The circles indicate individual sample values, and the straight line represents the correlation line. As 10 outcome measures were tested against one hypothesized predictor, to account for the increased possibility of type-I errors due to multiple testing, a Bonferroni-adjusted significance level of 0.005 (=0.05/10) was calculated.

**Figure 3:**
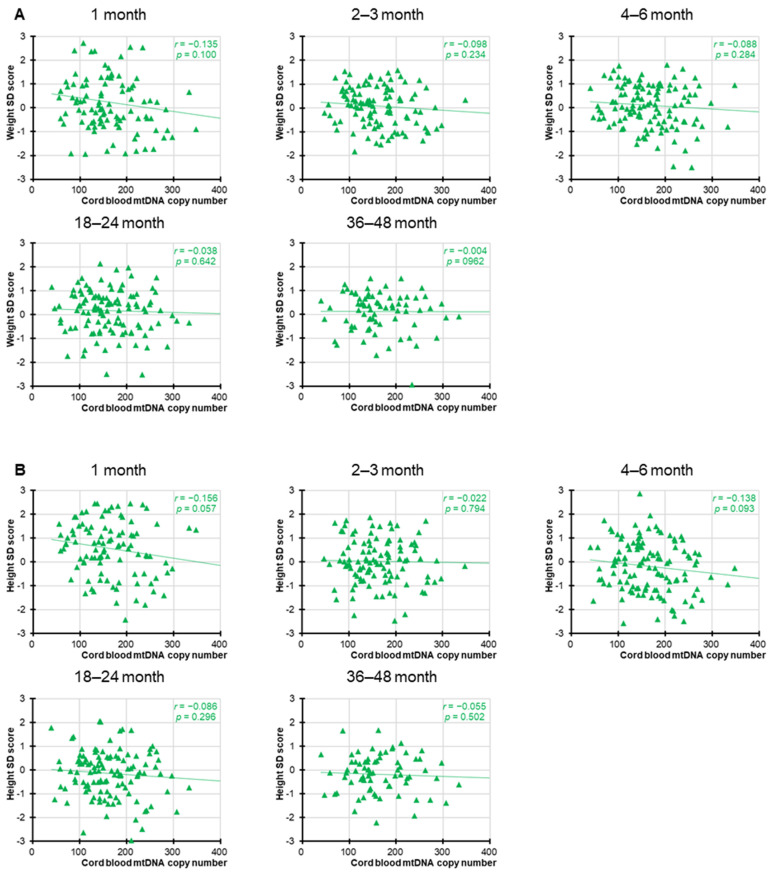
Correlation between female and male cord blood mtDNAcn and weight and height in infancy and toddlerhood. Correlation between all cord blood mtDNAcn and weight (**A**) and height (**B**) 1, 2–3, 4–6, 18–24, and 36–48 months after birth. The circles indicate individual sample values, and the straight line represents the correlation line. As 10 outcome measures were tested against one hypothesized predictor, to account for the increased possibility of type-I errors due to multiple testing, a Bonferroni-adjusted significance level of 0.005 (=0.05/10) was calculated.

Sex differences in weight and height between male and female infants were examined using Student’s *t*-test (Table 1). Normality of mtDNAcn distribution was assessed using a chi-square goodness-of-fit test, and no significant deviation from a normal distribution was observed. Therefore, Pearson’s correlation coefficient was applied to assess the relationships between the two variables (Figure 1, Figure 2 and Figure 3). Values greater than ±3 SD were excluded as outliers. Statistical significance was set at *p*  <  0.05 (two-sided) in a continuous model. To account for the increased possibility of type-I errors due to multiple testing, we used Bonferroni correction to adjust the significance level.

## 3. Results

Table 1 summarizes the mean ± SD of weight and height SD scores in female and male newborns at birth and at five postnatal time points: 1, 2–3, 4–6, 18–24, and 36–48 months. As values greater than ±3 SD were excluded as outliers, a total of 150 newborns were initially enrolled (68 females and 82 males), but one male newborn was excluded based on this criterion, resulting in 81 males included in the analysis. The sample sizes for each time point are also provided.

A significant difference in height SD scores was observed between male and female infants at birth (*p* = 0.002), whereas no significant differences were found at any of the other postnatal time points. At the 36–48-month follow-up, data were available for 54.4% of the female participants (37 out of 68) as well as 47.6% of the male (39 out of 82).

Significant negative correlations between cord blood mtDNAcn and birth parameters, including birth weight, length, and head circumference, were observed, as previously reported [19]. However, as shown in Figure 1 and Figure 2, in the present study, no statistically significant associations were found between cord blood mtDNAcn and the SD scores for weight or height at any of the postnatal time points for either sex. Nominally significant associations were observed for female height at 1 month (*r* = −0.272, *p* = 0.025) and male height at 36–48 months (*r* = −0.225, *p* = 0.042), but neither remained significant after Bonferroni correction (adjusted significance threshold *p* < 0.005). In addition, we performed a supplementary analysis combining both sexes into a single group (Figure 3). While this approach increased the overall statistical power, the results remained broadly consistent and showed no meaningful differences across analyses.

To provide additional detail on the postnatal growth trajectories of participants with extreme cord blood mtDNAcn values, we plotted longitudinal weight-for-age and height-for-age SD scores for individuals in the lowest (≤10th percentile) and highest (≥90th percentile) mtDNAcn groups. These individual-level trajectories are shown in Figures S1 (females) and S2 (males). Each line in the graphs represents the SD score changes over time for one subject at five postnatal time points (1, 2–3, 4–6, 18–24, and 36–48 months). In both sexes, the weight and height SD scores in the low and high mtDNAcn groups exhibited diverse individual patterns. No consistent trend in growth directionality was observed across the group, and most trajectories appeared to converge toward the population mean over time. These descriptive patterns support the quantitative findings that no statistically significant association was found between cord blood mtDNAcn and anthropometric outcomes at the postnatal time points assessed.

## 4. Discussion

Mitochondria play a central role in energy production and redox balance during embryonic and fetal development. Also, mtDNAcn is tightly regulated by nuclear-encoded transcriptional coactivators such as TFAM [22], which respond to changes in cellular energy demand and redox status. This regulation is especially critical in metabolically active tissues such as the brain, heart, and skeletal muscle. Disruption in these regulatory pathways may not manifest immediately but could predispose individuals to subtle mitochondrial dysfunction later in life. Thus, within the framework of the DOHaD hypothesis, cord blood mtDNAcn, a potential biomarker of fetal environmental exposure, has been reported to reflect intrauterine conditions and has shown associations with various perinatal outcomes, including birth weight and length [19]. These observations indicate that mtDNAcn may serve as an indicator of fetal metabolic adaptation and mitochondrial function at birth.

To our knowledge, this is the first study to investigate the correlation between cord blood mtDNAcn and physical growth aged 1 month to 36–48 months. According to the results, although cord blood mtDNAcn was negatively correlated with birth weight, this correlation seemed to disappear over time. In addition, there was no obvious relationship between cord blood mtDNAcn and physical growth in infancy and toddlerhood, even when focusing on the low and high cord blood mtDNAcn groups. We note that a nominally significant association was observed for female height at 1 month and male height at 36–48 months, but both findings disappeared after Bonferroni correction and should therefore be interpreted with caution. Postnatal environmental factors, such as nutritional status, may have a greater influence on physical growth during infancy and toddlerhood than cord blood mtDNAcn, as height and weight trajectories tend to stabilize in the months following birth. Although the small sample size is a limitation of the study, these results contribute to understanding of the physiological significance of cord blood mtDNAcn in physical growth.

The lack of association in the postnatal period may be attributable to the increasing influence of external environmental and behavioral factors, such as nutrition, physical activity, infection, and socioeconomic status, on physical development after birth. At the same time, it should be acknowledged that our analyses did not quantify or adjust for maternal and perinatal background variables (e.g., maternal age, body mass index, and smoking status) or for key postnatal covariates, including breastfeeding, nutritional intake, and caregiving practices, which are well-established determinants of infant growth [23,24], as well as parental genetic influences. Furthermore, parental socioeconomic background may also influence postnatal growth trajectories, as suggested by a recent systematic review, although the available evidence remains limited and heterogeneous [25]. The absence of these data represents an important limitation of our study, as such unmeasured factors could have acted as confounders and potentially attenuated or masked the association between cord blood mtDNAcn and postnatal growth. Therefore, our null findings should be interpreted with caution, as they do not exclude the possibility that true associations exist but were not detectable due to these limitations. This pattern may instead reflect the complex interplay between prenatal programming and postnatal environmental influences that together shape developmental trajectories. The relatively small sample size may have limited the statistical power to detect modest associations. In addition, several unmeasured covariates such as maternal nutrition, socioeconomic background, and other environmental factors represent major potential confounders that could have influenced both mtDNAcn and postnatal growth. Future studies with larger sample sizes and more comprehensive adjustment for these factors will be essential to clarify the true nature of these relationships.

In addition, because anthropometric measurements were obtained at broad time intervals and SD scores were used, we were unable to evaluate growth velocity (e.g., weight or height gain per unit time), which may provide complementary insights by accounting for interindividual variability at birth. Growth velocity is considered a more sensitive indicator of developmental dynamics, reflecting not only absolute size but also the tempo of postnatal growth. However, the sparse timing of measurements in the present dataset precluded such longitudinal modeling. Future studies with more frequent follow-up assessments would enable a more detailed evaluation of growth trajectories and their relationship with cord blood mtDNAcn.

Recent evidence has strengthened the notion that cord blood mtDNAcn may serve as a marker of future metabolic health. Reddam et al. reported an inverse association between cord blood mtDNAcn and childhood adiposity, suggesting that lower mtDNAcn at birth may increase the risk of early-life obesity [26]. Beyond mitochondrial indicators, other molecular biomarkers have also been extensively studied within the DOHaD framework. Epigenetic mechanisms, particularly DNA methylation, have been investigated in birth cohorts as mediators linking prenatal exposures to later health outcomes [27], and Bianco-Miotto et al. reviewed how early-life exposures can shape gene regulation through epigenetic modifications [28]. Consistently, a recent epigenome-wide association study demonstrated that newborn mtDNAcn was associated with DNA methylation patterns that persisted into childhood and were further related to cognitive development, highlighting a potential epigenetic pathway through which mitochondrial state at birth may influence later outcomes [29]. In addition, telomere length has been proposed as another biomarker of developmental programming; for example, an Australian cohort study found that adverse pregnancy outcomes were associated with shorter telomere length at 17 years of age [30]. These findings indicate that mtDNAcn should be interpreted in the broader context of molecular biomarkers used to investigate the underlying mechanisms of DOHaD. Although our study did not observe associations between mtDNAcn and postnatal anthropometric measures such as weight and height, this contrast underscores the importance of investigating other developmental outcomes. In particular, metabolic traits may be more sensitive to mitochondrial influences during the fetal period and could provide further insight into early-life programming mechanisms.

The participants in the TMM BirThree Cohort Study continue to be followed up, providing further epidemiological data on growth, biological aging, and the incidence of various diseases, including lifestyle-related ones. Given that many chronic diseases related to mitochondrial dysfunction emerge later in life, extended follow-up of this cohort into adolescence and adulthood will be essential. Such longitudinal data may help reveal latent associations between early-life mitochondrial states and long-term health outcomes, including cardiometabolic diseases, neurodevelopmental disorders, and aging-related phenotypes. In particular, as additional clinical data become available, future analyses should examine the relationships between cord blood mtDNAcn and cardiometabolic risk factors such as blood pressure, lipid profiles, and diabetes, which may provide further insight into the developmental origins of chronic disease. From the DOHaD perspective, further investigation is needed to clarify the physiological and pathological roles of mtDNAcn not only during childhood but also across the adult life course. Our findings reinforce the importance of incorporating molecular indicators such as mtDNAcn into long-term birth cohort studies, as these biomarkers may help bridge the gap between early-life biological states and later health outcomes. Although our findings did not reveal significant associations between cord blood mtDNAcn and postnatal growth, this study serves as an exploratory, hypothesis-generating pilot investigation. It provides a foundation for future research with larger cohorts and extended follow-up to better clarify the role of cord blood mtDNAcn in early-life programming.

## 5. Conclusions

In this cohort study of 150 newborns from the Tohoku Medical Megabank Birth and Three-Generation Cohort, cord blood mtDNAcn was not significantly associated with postnatal growth in weight or height up to 48 months of age. Growth trajectories of infants with higher or lower mtDNAcn values at birth tended to converge toward the population mean, suggesting that postnatal physical development is more strongly shaped by environmental and nutritional factors than by mitochondrial status at birth.

Although exploratory, these findings contribute to understanding the potential role of mtDNAcn within the framework of the Developmental Origins of Health and Disease (DOHaD) theory. The absence of significant associations highlights the importance of considering multiple biological, environmental, and social determinants when evaluating child growth. Future research with larger cohorts, comprehensive assessment of postnatal exposures, and extended follow-up into later life stages will be essential to clarify the relevance of cord blood mtDNAcn for pediatric growth, metabolic outcomes, and long-term health trajectories.

## Figures and Tables

**Table 1:** Changes in weight and height SD scores from birth to 36–48 months.

Characteristics	Female		Male		*p* Value
	***n***	**Mean ± SD**	***n***	**Mean ± SD**	
**Birth**					
Weight SDS score	68	0.31 ± 0.90	81	0.41 ± 0.94	0.484
Height SDS score	68	0.16 ± 0.97	81	0.63 ± 0.89	0.002
**1 month**					
Weight SDS score	48	0.15 ± 1.05	53	0.30 ± 1.42	0.572
Height SDS score	48	0.61 ± 1.11	52	0.51 ± 1.20	0.676
**2–3 month**					
Weight SDS score	48	0.08 ± 0.89	60	0.08 ± 0.77	0.998
Height SDS score	47	0.20 ± 0.95	60	−0.10 ± 0.92	0.104
**4–6 month**					
Weight SDS score	57	0.17 ± 0.89	61	0.03 ± 0.92	0.419
Height SDS score	57	−0.12 ± 1.12	61	−0.24 ± 1.00	0.546
**18–24 month**					
Weight SDS score	56	0.24 ± 0.85	60	0.09 ± 0.95	0.373
Height SDS score	55	−0.15 ± 0.93	60	0.15 ± 1.04	0.993
**36–48 month**					
Weight SDS score	36	0.11 ± 0.74	39	0.14 ± 0.91	0.900
Height SDS score	37	−0.17 ± 0.79	38	−0.19 ± 0.94	0.952

## Data Availability

All data are available in the main text or the Supplementary Materials.

## Supplementary Materials

The following supporting information can be downloaded at https://www.mdpi.com/article/10.3390/children12101369/s1, Figure S1: Postnatal changes over time in the heights and weights of females with cord blood mtDNAcn percentile 10th or lower and 90th or higher; Figure S2: Postnatal changes over time in the heights and weights of males with cord blood mtDNAcn percentile 10th or lower and 90th or higher.

## Abbreviations

The following abbreviations are used in this manuscript:

mtDNA	mitochondrial DNA
nDNA	nuclear DNA
mtDNAcn	mitochondrial DNA copy number
DOHaD	Developmental Origins of Health and Disease
TMM BirThree Cohort Study	Tohoku Medical Megabank Project Birth and Three-Generation Cohort Study
ToMMo	Tohoku Medical Megabank Organization
IMM	Iwate Tohoku Medical Megabank Organization
SD	standard deviation
