# Supplementary material for: Cord Blood Mitochondrial DNA Copy Number and Physical Growth in Infancy and Toddlerhood: A Birth Cohort Analysis
Source: Children (Basel). 2025 Oct 10;12(10):1369. doi: 10.3390/children12101369 (PMC12563496; doi:10.3390/children12101369)
Supplement: Supplementary file 1 [file children-12-01369-s001.zip › children-3898454-supplementary.pdf]

*Supplementary materials for*

# Cord blood mitochondrial DNA copy number and physical growth in infancy and toddlerhood: A birth cohort analysis

**Hisanori Fukunaga Takeshi Yamaguchi, Hiroyoshi Iwata, and Atsuko Ikeda**

**A** Percentile 10th and under

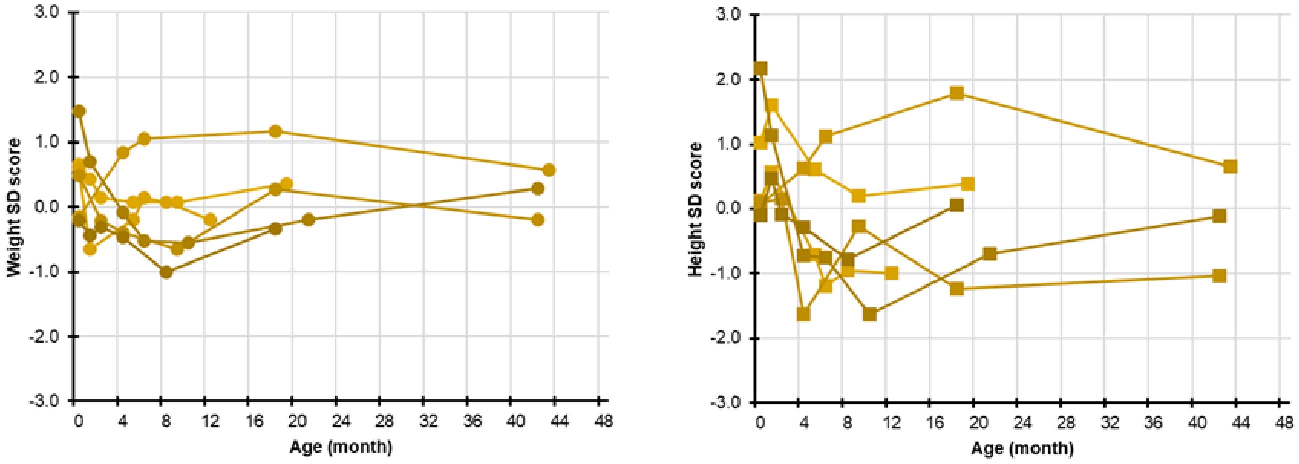


**B** Percentile 90th and above

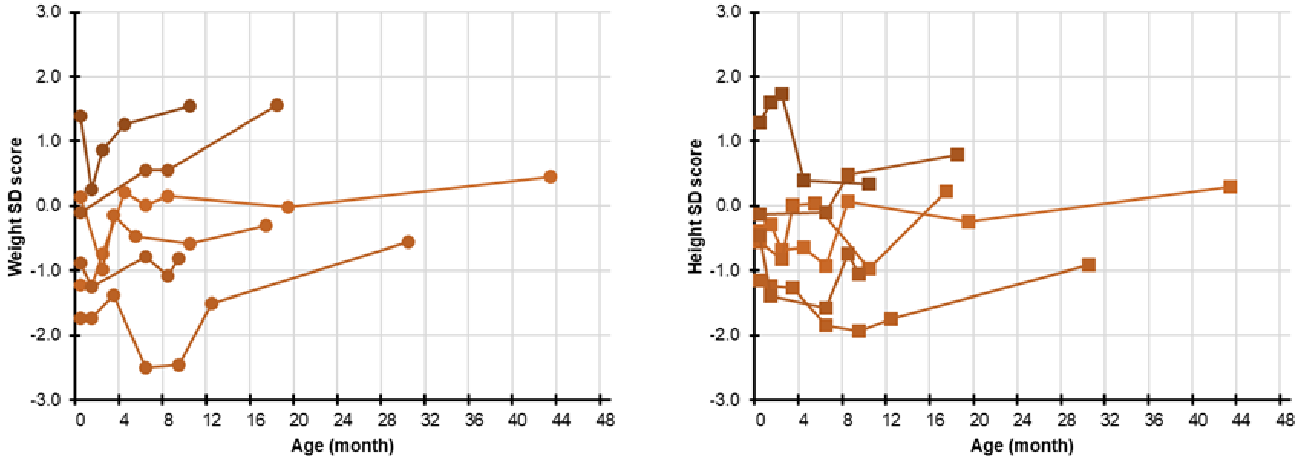


**Figure S1.** Postnatal changes over time in the heights and weights of females with cord blood mtDNAcn percentile 10th or lower and 90th or higher. Each line in the graph represents the change over time for one subject.

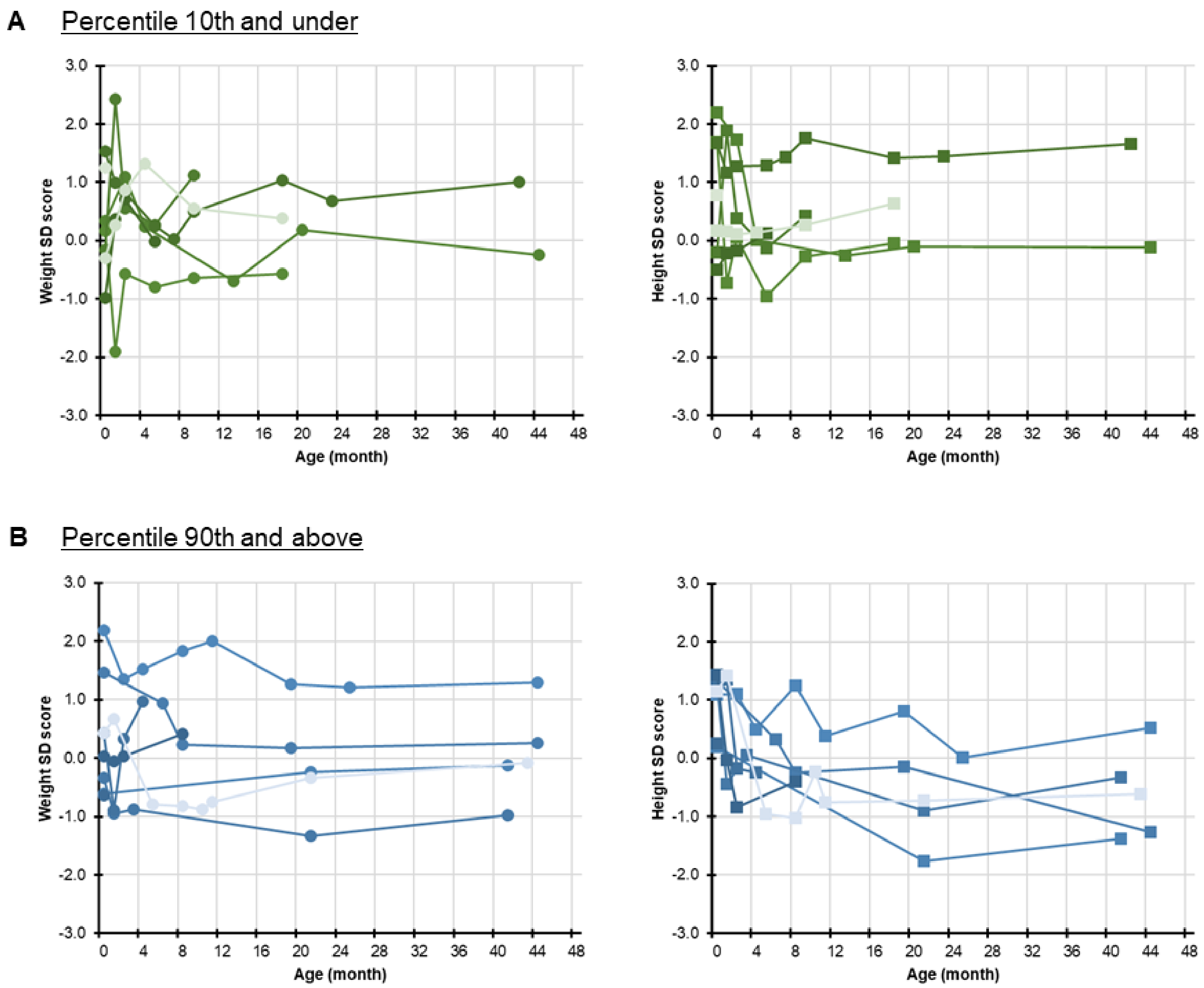


**Figure S2.** Postnatal changes over time in the heights and weights of males with cord blood mtDNAcn percentile 10th or lower and 90th or higher. Each line in the graph represents the change over time for one subject.
